# BMP signaling suppresses *Gemc1* expression and ependymal differentiation of mouse telencephalic progenitors

**DOI:** 10.1038/s41598-020-79610-6

**Published:** 2021-01-12

**Authors:** Hanae Omiya, Shima Yamaguchi, Tomoyuki Watanabe, Takaaki Kuniya, Yujin Harada, Daichi Kawaguchi, Yukiko Gotoh

**Affiliations:** 1 Graduate School of Pharmaceutical Sciences, The University of Tokyo, 7-3-1 Hongo, Bunkyo-ku, Tokyo, 113-0033 Japan; 2 International Research Center for Neurointelligence (WPI-IRCN), The University of Tokyo, 7-3-1 Hongo, Bunkyo-ku, Tokyo, 113-0033 Japan

**Keywords:** Neural stem cells, Development of the nervous system

## Abstract

The lateral ventricles of the adult mammalian brain are lined by a single layer of multiciliated ependymal cells, which generate a flow of cerebrospinal fluid through directional beating of their cilia as well as regulate neurogenesis through interaction with adult neural stem cells. Ependymal cells are derived from a subset of embryonic neural stem-progenitor cells (NPCs, also known as radial glial cells) that becomes postmitotic during the late embryonic stage of development. Members of the Geminin family of transcriptional regulators including GemC1 and Mcidas play key roles in the differentiation of ependymal cells, but it remains largely unclear what extracellular signals regulate these factors and ependymal differentiation during embryonic and early-postnatal development. We now show that the levels of Smad1/5/8 phosphorylation and Id1/4 protein expression—both of which are downstream events of bone morphogenetic protein (BMP) signaling—decline in cells of the ventricular-subventricular zone in the mouse lateral ganglionic eminence in association with ependymal differentiation. Exposure of postnatal NPC cultures to BMP ligands or to a BMP receptor inhibitor suppressed and promoted the emergence of multiciliated ependymal cells, respectively. Moreover, treatment of embryonic NPC cultures with BMP ligands reduced the expression level of the ependymal marker *Foxj1* and suppressed the emergence of ependymal-like cells. Finally, BMP ligands reduced the expression levels of *Gemc1* and *Mcidas* in postnatal NPC cultures, whereas the BMP receptor inhibitor increased them. Our results thus implicate BMP signaling in suppression of ependymal differentiation from NPCs through regulation of *Gemc1* and *Mcidas* expression during embryonic and early-postnatal stages of mouse telencephalic development.

## Introduction

Ependymal cells are multiciliated epithelial cells that constitute the wall of the lateral ventricles in the adult mammalian brain. The coordinated beating of the cilia of these cells generates a flow of cerebrospinal fluid (CSF) that carries essential nutrients to and removes waste material from central nervous system (CNS) tissue^1–5^. Impairment of ependymal cell differentiation during development thus leads to brain malformation and disorders such as hydrocephalus^2,6–13^.

The subventricular zone (SVZ) of the lateral ventricles in adult mice is a neurogenic niche where neural stem cells (NSCs) produce neurons throughout life^14–16^. These NSCs and surrounding ependymal cells form a pinwheel architecture at the ventricular surface^17^, and the ependymal cells regulate the number and activity of the NSCs via secreted and membrane proteins and thereby control the rate of neurogenesis^18–21^. The flow of CSF promotes the migration of SVZ-derived newborn neurons to the olfactory bulb in an epithelial sodium channel dependent manner^22,23^, with ependymal cells therefore playing essential roles in adult neurogenesis.

Ependymal cells of the lateral ventricles are derived from a subpopulation of neural stem-progenitor cells (NPCs, also known as radial glial cells) that becomes postmitotic at the late embryonic stage of development [around embryonic day (E) 14 in mice]^24^. A specific subpopulation of NPCs that constitutes the embryonic origin of adult NSCs in the lateral SVZ also becomes postmitotic at E13 to E15 in mice^25,26^. Recent studies have shown that some ependymal cells and adult NSCs share a common origin at the late embryonic stage^27,28^. The Geminin family of cell cycle and transcriptional regulators—including Geminin, GemC1 (also known as Gmnc or Lynkeas), and Mcidas (Mci or Idas)—as well as the Forkhead family protein Foxj1 play pivotal roles in fate specification and differentiation of ependymal cells during development^28–34^. Both GemC1 and Mcidas have been shown to promote multiciliated cell differentiation in the brain and other organs (such as the airways and reproductive organs)^35–39^. Various gain- and loss-of function studies have revealed that GemC1 regulates early specification of multiciliated cells and acts upstream of Mcidas and Foxj1, whereas Mcidas promotes basal body amplification and maturation of multiciliated cells together with the transcription factors TAp73, c-Myb, E2F4, and E2F5^30,31,39–45^. In the context of embryonic NPCs in the SVZ, whereas overexpression of Geminin increases the number of clones that contain adult NSCs, that of GemC1 increases the number of those containing muticiliated ependymal cells, supporting the notion that GemC1 plays a key role in the early fate specification of ependymal cells^28^. Whereas Notch signaling, epidermal growth factor receptor (EGFR) signaling, and the extracellular matrix (ECM) protein dystroglycan have been shown to regulate the expression of Mcidas and Foxj1 during ependymal maturation^31,46–48^, little is known of the extracellular signals that may regulate the expression of GemC1 and thereby contribute to the specification and differentiation of ependymal cells during embryonic and early-postnatal telencephalic development.

Bone morphogenetic proteins (BMPs) belong to the transforming growth factor–β (TGF-β) superfamily of cytokines and are recognized by type I receptors (BMPR1A/ALK3, BMPR1B/ALK6, and ActR-I/ALK2) and type II receptors (BMPR-II/BMPR2, ActR-II, and ActR-IIB)^49^. BMP2 and BMP4 preferentially bind to ALK3 and ALK6, whereas BMP6 and BMP7 bind to ALK2 and, to a lesser extent, to ALK6^49^. Activation of these receptor serine-threonine kinases results in the phosphorylation and consequent activation of R-Smads (Smad1, Smad5, and Smad8), which, together with Co-Smad (Smad4), induce transcription of downstream target genes including those for the Id family of transcription factors^49^. BMPs perform a variety of functions during development of the vertebrate CNS, ranging from the induction of neuroectoderm and its dorsoventral patterning to regulation of the proliferation, survival, and neuronal and glial differentiation of NPCs^50^. In the adult mouse brain, BMP-Id signaling also contributes to the maintenance of quiescent NSCs^51–54^.

We here show that the levels of phosphorylated R-Smads and of Id proteins decline during the course of mouse telencephalic development in a manner that appears to coincide both temporally and spatially with the differentiation of ependymal cells. We found that forced activation or inhibition of BMP signaling suppressed or promoted multiciliated ependymal cell differentiation, respectively, in primary NPC cultures isolated from the embryonic and postnatal mouse telencephalon. We also found that BMP signaling suppressed the expression of *Gemc1*, *Mcidas*, and *Foxj1* genes as well as the differentiation of ependymal-like cells even in embryonic NPC cultures. Our results thus implicate BMP signaling in suppression of ependymal differentiation at the embryonic and early-postnatal stages of development of the mouse telencephalon.

## Results

### BMP signaling declines during SVZ development coincident with the initial phase of ependymal differentiation

**Figure 1:**
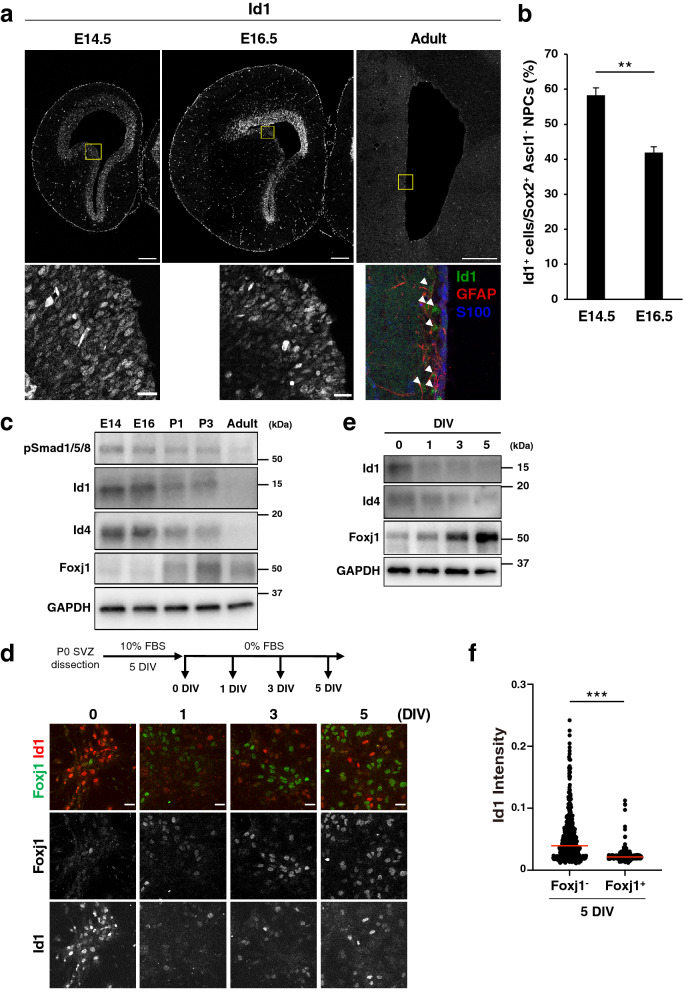
BMP signaling declines during ependymal cell differentiation. (**a**) Immunohistofluorescence analysis of Id1 in the LGE of the embryonic mouse brain at E14.5 or E16.5 as well as of Id1, glial fibrillary acidic protein (GFAP), and S100 in the SVZ of the lateral ventricles in the adult mouse brain (P56–P63). The boxed regions of the upper panels are shown at higher magnification in the corresponding lower panels. Arrowheads indicate Id1^+^ cells. Scale bars, 200 μm (upper panels) or 20 μm (lower panels). Data are representative of three independent experiments. (**b**) Quantification by immunohistofluorescence analysis of Id1^+^ cells among Sox2^+^Ascl1^–^ NPCs in the LGE at E14.5 and E16.5. Data are means ± SEM (n = 3 embryos for each stage). Three sections were counted per sample. Total of 500–800 cells were counted per section. **p < 0.01 (two-tailed Student’s unpaired *t* test). (**c**) Immunoblot analysis of the VZ-SVZ of the embryonic GE and corresponding postnatal basal ganglia at the indicated embryonic, postnatal, and young adult (P30) stages with antibodies to phosphorylated (p) Smad1/5/8, to Id1, to Id4, and to glyceraldehyde-3-phosphate dehydrogenase (GAPDH, loading control). Data are representative of three independent experiments, with each experiment analyzed several times. (**d**) Dissociated cells of the lateral ventricular wall of P0 mice were cultured first for 5 days in vitro (DIV) in the presence of 10% FBS (proliferation medium) and then for up to 5 DIV in the absence of serum (differentiation medium). The cells were subjected to immunocytofluorescence staining with antibodies to Foxj1 and to Id1 at the indicated times. Scale bars, 20 μm. Data are representative of three independent experiments. (**e**) Immunoblot analysis of cultures as in (**d**) with antibodies to Id1, to Id4, and to GAPDH. Data are representative of three independent experiments. (**f**) Id1 immunofluorescence intensity in Foxj1^+^ cells (n = 395) and Foxj1^–^ cells (n = 484) in cultures as in (**d**) analyzed at 5 DIV. Each dot represents one cell, and the red bar indicates the median. Data are from three independent experiments. ***p < 0.001 (two-tailed Student’s unpaired *t* test).

Id1 and Id4 proteins have been shown to maintain the quiescence of NSCs in the adult SVZ^52–54^. Only a minor fraction of cells in the adult mouse SVZ (presumably, adult NSCs) show a high level of Id1 expression, whereas S100^+^ ependymal cells show a low level of Id1 expression^52^ (Fig. 1a). We first asked whether the levels of Id1 and Id4 proteins are regulated during SVZ development. We found that Id1 immunofluorescence intensity varied among NPCs in the ventricular zone (VZ) of the lateral ganglionic eminence (LGE) even at E14.5 (Fig. 1a). Moreover, the overall level of Id1 immunostaining intensity within the VZ-SVZ of the embryonic LGE appeared to decrease from E14.5 to E16.5 (Fig. 1a). Indeed, the fraction of Id1^+^ cells among Sox2^+^ Ascl1^–^ NPCs declined from E14.5 to E16.5 (Fig. 1b). This down-regulation of Id1 thus precedes the maturation of S100β^+^ acetylated tubulin^+^ ependymal cells that takes place after birth^24,55^. To examine further the levels of Id proteins during development, we isolated the VZ-SVZ region of the embryonic LGE and corresponding postnatal basal ganglia and performed immunoblot analysis. The amounts of Id1 and Id4 declined between E16 and postnatal day (P) 1, apparently coinciding with the initial induction of Foxj1, a key regulator of ependymal differentiation (Fig. 1c, Sup. Figure 1)^29,33,34^. These results suggested that the overall decline in the abundance of Id proteins in the VZ-SVZ roughly coincides with the initial differentiation of ependymal cells in the developing basal ganglia.

Given that Id proteins are major effectors of BMP signaling, we asked whether the overall activity of BMP signaling in the VZ-SVZ also changes during development. Immunoblot analysis showed that the abundance of phosphorylated R-Smads (Smad1/5/8) also significantly decreased from E14 to P1 (Fig. 1c, Sup. Figure 1), supporting the notion that a decline in the level of BMP signaling coincides with the initial phase of ependymal differentiation.

We then established cultures of NPCs derived from the lateral ventricular wall at P0 according to a previously described protocol for the study of ependymal differentiation^56^. These NPCs were cultured first in the presence of 10% fetal bovine serum (FBS) and subsequently in the absence of serum, with serum removal inducing differentiation of the NPCs into multiciliated ependymal cells^46,56^. We found that the amount of Id1 protein, as well as that of Id4 protein to a lesser extent, in the cultured cells was reduced in response to serum removal, and that this effect appeared to coincide with the initial induction of Foxj1 (Fig. 1d, e, Sup. Figure 1). Moreover, the expression of Id1 and that of Foxj1 appeared to be mutually exclusive (Fig. 1d). Indeed, the intensity of Id1 immunoreactivity was significantly lower in Foxj1^+^ cells than in Foxj1^–^ cells at 5 days in vitro (DIV) after serum removal (Fig. 1f), further indicative of a negative correlation between BMP signaling and ependymal differentiation.

### BMP ligands suppress ependymal differentiation in postnatal NPC cultures

**Figure 2:**
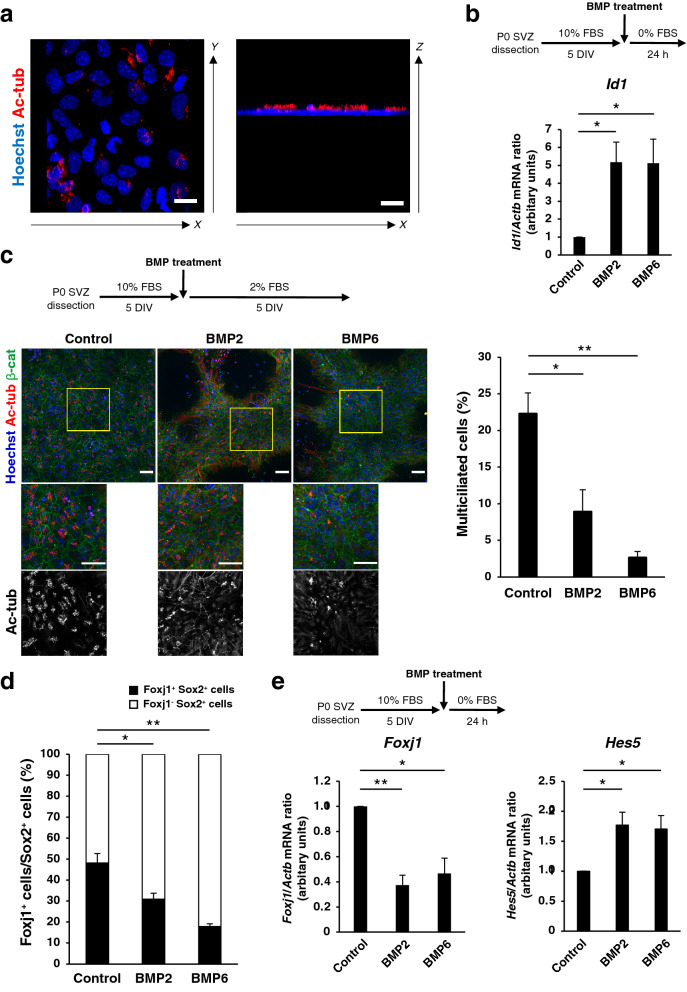
BMP signaling is sufficient for suppression of ependymal cell differentiation in postnatal NPC cultures. (**a**) P0 NPC cultures maintained for 5 days in the presence of 10% FBS and then for 5 days in the 2% FBS were subjected to immunocytofluorescence staining with antibodies to acetylated tubulin (Ac-tub). Nuclei were counterstained with Hoechst 33342. Immunofluorescence images in the z-projected *x*–*y* and y-projected *x*–*z* planes are shown. Scale bars, 20 μm. Data are representative of three independent experiments. (**b**) P0 NPC cultures were maintained for 5 days in the presence of 10% FBS and then for 24 h without serum but in the absence (Control) or presence of BMP2 (50 ng/ml) or BMP6 (50 ng/ml). The amount of *Id1* mRNA in the cells was then determined by quantitative RT-PCR analysis and normalized by the amount of *Actb* mRNA. Data are means ± SEM (n = 8 independent experiments). *p < 0.05 (Tukey’s test). (**c**) P0 NPC cultures maintained for 5 days in the presence of 10% FBS and then for 5 days with 2% FBS in the absence or presence of BMP2 (20 ng/ml) or BMP6 (20 ng/ml) were subjected to immunocytofluorescence staining with antibodies to Ac-tub and to β-catenin (β-cat, a marker of adherens juction). Nuclei were counterstained with Hoechst 33342. The boxed regions of the upper panels are shown at higher magnification in the corresponding middle and lower panels. Scale bars, 50 μm. Cells with bundles of Ac-tub were counted as multiciliated cells, and their percentage was determined. Data are means ± SEM (n = 3 independent experiments). *p < 0.05; **p < 0.01 (Tukey’s test). (**d**) Quantification by immunocytofluorescence analysis of Foxj1^+^ cells among Sox2^+^ NPCs for cultures treated as in (**c**). Three microscope fields were counted per sample. Total of 300–500 cells were counted per sample. Data are means ± SEM (n = 3 independent experiments). *p < 0.05; **p < 0.01 (Tukey’s test). (**e**) Quantitative RT-PCR analysis of *Foxj1* and *Hes5* mRNAs for cultures treated as in (**b**). Data are means ± SEM (n = 8 independent experiments). *p < 0.05, **p < 0.01 (Tukey’s test).

The removal or a reduction in the concentration of serum in the P0 NPC cultures results in ependymal differentiation over the course of several days, as is apparent from the generation of acetylated tubulin^+^ multiciliated cells^56^ (Fig. 2a). Given the apparent negative correlation between BMP signaling and ependymal differentiation, we examined whether BMP signaling actually affects ependymal differentiation by exposing the P0 NPC cultures to BMP2 or BMP6 at the time of serum removal. We confirmed that treatment with BMP2 or BMP6 increased the expression of the downstream target genes *Id1* and *Id4* (Fig. 2b and data not shown). BMP2 and BMP6 also each significantly reduced both the fraction of multiciliated cells detected at 5 DIV (Fig. 2c) as well as the fraction of Foxj1^+^ cells (Fig. 2d). Consistent with this result, treatment with BMP2 or BMP6 also resulted in significant down-regulation of the amount of *Foxj1* mRNA, whereas it significantly increased the expression of the NPC marker gene *Hes5* (Fig. 2e). These results thus suggested that BMP signaling suppresses the differentiation of NPCs into ependymal cells in this postnatal NPC culture system.

The reduced proportion of ependymal cells by the treatment with BMPs may be ascribable to its selective effects on proliferation or apoptosis of ependymal precursors. We found, however, that the fraction of cleaved caspase-3^+^ cells was very low (less than 5%) in this culture and that BMP treatment did not significantly change the proportion of cleaved caspase-3^+^ cells in both Foxj1^−^Sox2^+^ cells (NPCs) and Foxj1^+^Sox2^+^ (ependymal) cells (Sup. Figure 2). Moreover, BMP treatment did not increase, but rather reduced, the fraction of BrdU^+^ cells among Foxj1^-^Sox2^+^ cells, while having little effects on that among Foxj1^+^Sox2^+^ cells (Sup. Figure 3). These results support the notion that the reduction of ependymal cells by BMPs is not due to a selective increase of apoptosis or a selective reduction of proliferation of Foxj1^+^ ependymal precursors.

### A BMPR inhibitor promotes ependymal differentiation in postnatal NPC cultures

**Figure 3:**
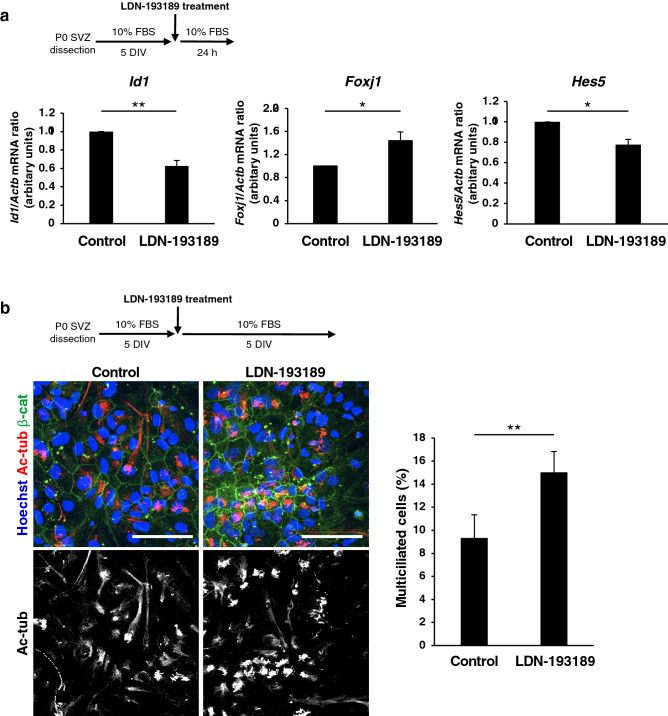
BMP signaling is necessary for suppression of ependymal cell differentiation in postnatal NPC cultures. (**a**) P0 NPC cultures were maintained for 5 days in the presence of 10% FBS and then for 24 h in the additional absence (Control) or presence of LDN-193189 (0.1 μM). The amounts of *Id1*, *Foxj1 *and *Hes5* mRNAs in the cells were then determined by quantitative RT-PCR analysis. Data are means ± SEM (n = 7 independent experiments). *p < 0.05; **p < 0.01 (two-tailed Student’s paired *t* test). (**b**) P0 NPC cultures maintained for 5 days in the presence of 10% FBS and then for 5 days in the additional absence or presence of LDN-193189 (0.1 μM) were subjected to immunocytofluorescence staining with antibodies to Ac-tub and to β-cat. Nuclei were counterstained with Hoechst 33342. Scale bars, 50 μm. Cells with bundles of Ac-tub were counted as multiciliated cells, and their percentage was determined. Three microscope fields were counted per sample. Total of 300–500 cells were counted per sample. Data are means ± SEM (n = 3 independent experiments). **p < 0.01 (two-tailed Student’s paired *t* test).

We then examined whether BMP signaling is required for ependymal differentiation in this culture system with the use of LDN-193189, an inhibitor of the BMP receptors ALK2 and ALK3. We confirmed that treatment of the NPC cultures with 0.1 μM LDN-193189 for 24 h in the presence of 10% FBS resulted in down-regulation of *Id1* expression (Fig. 3a). Such treatment also increased the level of *Foxj1* mRNA (Fig. 3a). Furthermore, treatment with LDN-193189 for 5 days resulted in a significant increase in the fraction of multiciliated cells (Fig. 3b), whereas that for 24 h induced a significant decrease in the expression of *Hes5* (Fig. 3a). These results suggested that BMP signaling is necessary for efficient suppression of ependymal differentiation in this postnatal NPC culture system.

### BMP ligands suppress ependymal cell-like differentiation in embryonic NPC cultures

**Figure 4:**
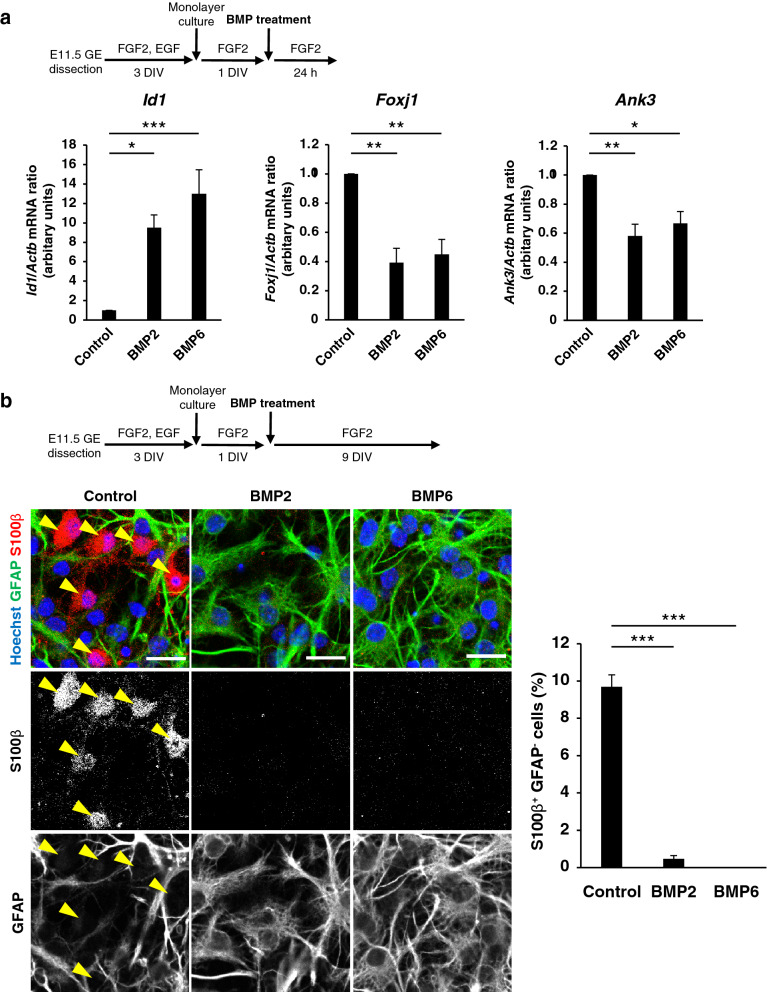
BMP signaling suppresses differentiation of ependymal-like cells among embryonic progenitors. (**a**) Cells dissociated from the GE of E11.5 embryos were cultured for 3 days in suspension, and the resulting neurospheres were dissociated and cultured as monolayers for 1 day before exposure to BMP2 (50 ng/ml) or BMP6 (50 ng/ml) for 24 h. The amounts of *Id1*, *Foxj1* and *Ank3* mRNAs in the cells were then determined by quantitative RT-PCR analysis. Data are means ± SEM (n = 3 independent experiments). *p < 0.05; **p < 0.01; ***p < 0.001 (Tukey’s test). (**b**) Cultures as in (**a**) exposed to BMP2 or BMP6 for 9 days (with replenishment of the medium every 2 days) were subjected to immunocytofluorescence staining with antibodies to S100β and to GFAP. Nuclei were counterstained with Hoechst 33342. Arrowheads indicate S100β^+^GFAP^–^ ependymal-like cells. Scale bars, 20 μm. The percentage of S100β^+^GFAP^–^ cells was determined. Three microscope fields were counted per sample. Total of 200–300 cells were counted per sample. Data are means ± SEM (n = 3 independent experiments). ***p < 0.001 (Tukey’s test).

The down-regulation of Id proteins apparent in the VZ-SVZ at the late embryonic stage of LGE development (Fig. 1a) prompted us to examine the possible role of BMP signaling in the initial stage of ependymal cell differentiation. We therefore isolated NPCs from the GE at E11.5 and cultured them first for 3 days in suspension and then as monolayers. Exposure of these cells to BMP2 or BMP6 for 24 h at 1 day after the onset of monolayer culture resulted in a significant decrease in the expression of *Foxj1* and *Ank3*, which encodes an effector of Foxj1 responsible for the lateral adhesion of ependymal cells^56^, as well as a significant increase in that of *Id1* (Fig. 4a). After 10 days of monolayer culture, a fraction of control cells had initiated expression of S100β, indicative of ependymal differentiation, without expression of the astrocyte marker GFAP (Fig. 4b). Treatment with BMP2 or BMP6 markedly suppressed this increase in the number of S100β^+^GFAP^–^ cells (Fig. 4b). These results suggested that BMP signaling suppresses ependymal cell-like differentiation even at an early stage of development equivalent to the perinatal stage. We also observed that exposure of this embryonic NPC culture to BMP2 or BMP6 increased the fraction of GFAP^+^ cells and reduced that of BrdU^+^ cells among Sox2^+^ cells (Sup. Figure 4). This is consistent with the previous notion that BMP treatment promotes establishment of “quiescent” NSCs^51,57^, while suppressing ependymal differentiation.

### BMP ligands suppress expression of *Gemc1* and *Mcidas*

**Figure 5:**
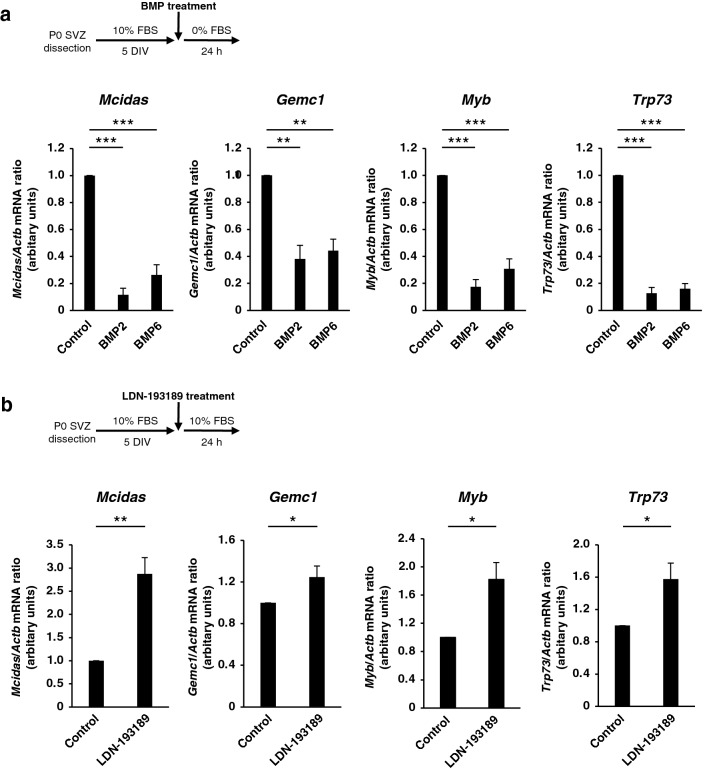
BMP signaling suppresses the expression of initial key regulators of ependymal cell differentiation. (**a**) P0 NPC cultures maintained for 5 days in the presence of 10% FBS and then for 24 h in serum-free medium containing BMP2 (50 ng/ml) or BMP6 (50 ng/ml) were subjected to quantitative RT-PCR analysis for determination of the amounts of *Mcidas*, *Gemc1*, *Myb*, and *Trp73* mRNAs. Data are means ± SEM (n = 8 independent experiments). **p < 0.01, ***p < 0.001 (Tukey’s test). (**b**) P0 NPC cultures maintained for 5 days in the presence of 10% FBS and then for 24 h in the additional absence or presence of LDN-193189 (0.1 μM) were analyzed as in (**a**). Data are means ± SEM (n = 7, 10, 7, or 6 independent experiments, respectively). *p < 0.05; **p < 0.01 (two-tailed Student’s paired *t* test).

Given that Geminin family members play a central role in the initial steps of ependymal differentiation, we next asked whether BMP signaling regulates the expression of the corresponding genes. The amount of *Mcidas* mRNA was significantly reduced by exposure of P0 NPC cultures to BMP2 or BMP6 for 24 h after serum removal (Fig. 5a). Moreover, the amounts of mRNAs for *Myb* (which encodes c-Myb) and *Trp73* (which encodes TAp73), which act upstream of *Foxj1*, were also reduced by such BMP treatment (Fig. 5a). Importantly, the expression of *Gemc1*, which acts upstream of *Mcidas*, was significantly suppressed by BMP2 or BMP6 (Fig. 5a). Conversely, the levels of *Mcidas* and *Gemc1* mRNAs as well as those of *Myb* and *Trp73* mRNAs were increased by treatment of the cultures with the BMP receptor inhibitor LDN-193189 for 24 h in the continued presence of serum (Fig. 5b). Together, these results implicated BMP signaling in suppression of the key initial regulators of ependymal cell differentiation.

## Discussion

Although the transcriptional network that governs multiciliated cell differentiation in various organs has been well characterized, it has remained unclear how extrinsic signals regulate this network in the niche that supports ependymal cell specification and differentiation during telencephalic development. Here we show that BMP signaling suppresses the differentiation of mouse GE-derived NPCs into ependymal cells. We thus detected a reduction in the level of BMP signaling (as indicated by the abundance of phosphorylated Smad1/5/8 and of Id proteins) in the VZ-SVZ of the lateral ventricles that coincided with the initial specification and differentiation of ependymal cells. Mutually exclusive expression patterns of Id1 and Foxj1 in NPC cultures also supported a negative role for BMP signaling in ependymal specification. Indeed, we found that BMP signaling suppressed the expression of *Gemc1*, a key regulator of ependymal specification, as well as that of *Mcidas* and *Foxj1*, master regulators of ependymal differentiation (basal body amplification). These results implicate BMP signaling in negative regulation of the specification and differentiation of ependymal cells in the embryonic GE and early postnatal basal ganglia of the mouse telencephalon. BMP signaling has previously been shown to negatively regulate the differentiation of multiciliated cells in the mucociliary epithelium of airways as well as in embryonic stem cell cultures^58,59^, although the molecular mechanisms underlying this regulation remain unclear. It would thus be of interest to investigate whether BMP signaling plays a similar role in suppression of *Gemc1* and *Mcidas* expression in the differentiation of other multiciliated cells.

Possible sources of BMP ligands in the regulation of NPCs include CSF, blood vessels, and NPCs themselves. The mouse choroid plexus, which releases factors into CSF, expresses BMP ligands including BMP2, BMP4, BMP5, and BMP7^5,60^. A proteomics analysis detected the presence of BMP6 and BMP7 in human CSF^61^. Endothelial cells also express BMP2 and BMP4^62^, and NPCs in the LGE express BMP6 at the late embryonic stage (data not shown). It is thus plausible that NPCs are exposed to BMP ligands in the VZ-SVZ of the developing LGE. How then do NPCs escape from these BMP ligands in order to differentiate into ependymal cells? More specifically, what mechanisms may underlie the decline in the level of BMP signaling we detected at the VZ-SVZ of the lateral ventricles when NPCs differentiate into ependymal cells? One such mechanism may involve the secretion of BMP inhibitor proteins such as Noggin and low density lipoprotein–related protein 2 (LRP2), both of which are expressed in adult ependymal cells and antagonize the effects of BMP ligands so as to allow the activation of NSCs and the production of neurons in the adult SVZ^19,63^. The expression of Noggin or LRP2 in a subpopulation of embryonic NPCs may support the formation of a niche in which BMP signaling is locally suppressed so as to promote ependymal specification and differentiation.

Ependymal cells and adult NSCs have recently been shown to share a common embryonic origin, with GemC1 and Geminin being respective determinants of these lineages^28^. It remains unknown, however, how the GemC1^+^ ependymal lineage and the Geminin^+^ adult NSC lineage are segregated from common NPCs during development. Given that we now show that BMP signaling inhibits *Gemc1* expression in NPCs, BMP signaling might control this bifurcation process, although this hypothesis should be proven by clonal analyses of NPCs in future studies. If this is the case, it would be of interest to investigate how differential sensitivity to BMP ligands might emerge among NPCs. For instance, differential expression of BMP inhibitors or BMP signaling components might underlie segregation between ependymal and adult NSC lineages.

What might be the direct target (or targets) of BMP signaling in the regulation of ependymal differentiation? Given that the *Gemc1* locus contains multiple Smad binding elements (SBEs), and that ChIP Atlas data indicate that Smad4 binds to this locus in mouse NS5 neural stem cells^64^, it is possible that *Gemc1* is a direct target of activated Smad proteins in the BMP signaling pathway, although R-Smads usually function as positive regulators of transcription. Alternatively, Id proteins (effectors of Smad proteins and negative regulators of transcription) may regulate genes related to ependymal differentiation, in which case, given their roles in adult NSC maintenance^52–54^, Id proteins may act at the lineage bifurcation between ependymal cells and adult NSCs.

Ependymal cells and postnatal-adult NSCs form a distinct pinwheel structure at the ventricular surface after their differentiation from NPCs^17^. Of note in this regard, BMP treatment of postnatal NPC cultures reproducibly induced marked changes in cellular architecture that generated gaps in the monolayer culture (Fig. 2c, lower magnification images). This effect may be due to differential lateral cell adhesion^56^ and the sorting of ependymal cells and other cell types induced by the addition of BMP ligands. In addition, reorganization of ECM might contribute to this process, given that dystroglycan-mediated ECM reorganization promotes postnatal maturation of ependymal cells (in part through control of Notch signaling and Mcidas and Foxj1 expression)^20,47^. It would thus be of interest to investigate how ECM reorganization and BMP signaling might interact during ependymal differentiation and formation of the pinwheel structure at the ventricular wall.

In conclusion, we have here uncovered a previously unappreciated role of BMP signaling in suppression of ependymal differentiation from NPCs, with this function of BMP signaling likely being mediated through regulation of the expression of the Geminin family members GemC1 and Mcidas as well as that of Foxj1. Abnormal ependymal differentiation is associated with brain malformation and disorders such as hydrocephalus^2,6–13,65–67^. Of interest, mice lacking the *Msx* gene, an established downstream target of BMP signaling in other systems, also develop hydrocephalus^59^. Our findings may thus provide insight into the development of such ependymal cell-related disorders.

## Experimental procedures

### Ethics statement

All animals were maintained and studied according to protocols approved by the Animal Care and Use Committee of The University of Tokyo (approval numbers: P25-8, P25-27, PH27-3 and P30-4). All procedures were followed in accordance with the University of Tokyo guidelines for the care and use of laboratory animals.

### Animals

Slc:ICR (ICR) and C57BL/6NCrSlc (B6J) mice were obtained from SLC Japan and were maintained in a temperature (20–26 °C)—and relative humidity (35–65%)—controlled environment with a normal 12‐h-light,12‐h-dark cycle. They were housed two to six per sterile cage (Innocage, Innovive) containing bedding chips (Palsoft, Oriental Yeast) and were provided with irradiated food (CE2, CLEA Japan) and filtered water ad libitum. Mouse embryos were isolated at various ages, with E0.5 being considered the time of vaginal plug appearance.

### Postnatal NPC culture

The differentiation of ependymal cells from isolated postnatal NPCs in culture was studied according to a modified version of a method described previously^56^. The lateral ventricular wall was dissected from Slc:ICR mice at P0, when NPCs are still bipotent that can produce both ependymal cells and glial cells^27,29^. Dissociated cells were transferred to poly-D-lysine (Sigma-Aldrich)–coated 24 well plate and cultured at a density of 1 × 10^6^ cells/ml in a proliferation medium consisting of Dulbecco’s modified Eagle’s medium containing high glucose (DMEM-High Glucose 4.5, Gibco) and supplemented with 10% FBS (Gibco) and 1% penicillin–streptomycin (Invitrogen). After culture for 5 days, the medium was switched to a differentiation medium (proliferation medium containing 0% or 2% FBS) supplemented (or not) with recombinant human/mouse/rat BMP2 or mouse BMP6 (R & D Systems) at 20 ng/ml or 50 ng/ml. BMP2 and BMP6 were dissolved in a solution containing 4 mM HCl and 0.1% bovine serum albumin, which was also used as a vehicle control. Alternatively, after culture for 5 days in proliferation medium, the cells were exposed to LDN-193189 (Sigma-Aldrich) at 0.1 μM in proliferation medium. LDN-193189 was dissolved in distilled water. For cell proliferation analysis, 10 μM 5-Bromo-2´-Deoxyuridine (BrdU, Invitrogen) was administered to the culture 2 h before sample collection.

### Embryonic NPC culture

Primary NPCs were isolated from the GE of Slc:ICR mouse embryos at E11.5. The dissected tissue was subjected to enzymatic digestion with a papain‐based solution (Sumitomo Bakelite), and the dissociated cells were cultured in DMEM-F12 (1:1, v/v) (Gibco) supplemented with B27 (Invitrogen), recombinant human fibroblast growth factor 2 (FGF2, 20 ng/ml) (Invitrogen) and recombinant human epidermal growth factor (EGF, 20 ng/ml) (Invitrogen). For cell proliferation analysis, 10 μM BrdU (Invitrogen) was administered to the culture 2 h before sample collection.

### Quantitative RT-PCR analysis

Total RNA was isolated from cell cultures with the use of RNAiso Plus (Takara), and portions (1 µg) of the RNA were subjected to RT with ReverTra Ace qPCR RT Master Mix with gDNA Remover (Toyobo). The resulting cDNA was subjected to real-time PCR analysis in a LightCycler 480 instrument (Roche) with KAPA SYBR fast qPCR Mix (Toyobo). Primer sequences (5′ → 3′, and forward and reverse, respectively) were as follows: *Actb*, ATAGTCATTCCAAGTATCCATGAAA and GCGACCATCCTCCTCTTAG; *Foxj1*, GGGCGAAATGGTCTCTAAG and GTCAGGCTGGAAGGTTTGTA; *Id1*, TACGACATGAACGGCTGCTA and TCTCCACCTTGCTCACTTT; *Gemc1*, TGGTCTCCTGGACAACACTG and TAACTCAGAGGGCGATTCCA; *Mcidas*, AACCGAAGCGTCTCCTAGTG and GGTCATCCATTGCATCTCTG; *Ank3*, CATCCTAAACTTCAAGTCCACACTATAA and AACTCACACAGTGGTGGTAA; *Trp73*, GCACCTACTTTGACCTCCCC and GCACTGCTGAGCAAATTGAAC; *Myb*, CTTGCAGCTCAAGAAATTAAATACG and ATCCGATTCCTGCTTAATCAC; and *Hes5*, AAGTGACTTCTGCGAAGTT and AAGTCCTCTACGGGCTG.

### Immunocytofluorescence analysis

Immunocytofluorescence analysis was performed as described previously^68^. Cells were fixed with 4% paraformaldehyde in phosphate buffered-saline (PBS), permeabilized for 10 min with 0.2% Triton X‐100 in PBS, exposed for 30 min to 3% bovine serum albumin in PBS (blocking solution), incubated first overnight at 4 °C with primary antibodies diluted in the blocking solution and then for 1 h at room temperature with Alexa Fluor–labeled secondary antibodies (1:1000 dilution) (A21206, A21202, A11039, A31572, A31570, A31571, A31573, A32795, A21447, Molecular Probes) and Hoechst 33342 (1:1000 dilution) (Molecular Probes) in blocking solution, and then mounted in Mowiol (Calbiochem). Primary antibodies and their dilutions were as follows: anti–acetylated α-tubulin, 1:500 (T6793, Sigma-Aldrich); anti-Id1, 1:500 (BCH-1/37-2, Biocheck); anti-β-catenin, 1:200 (C2206, Sigma-Aldrich); anti-Foxj1, 1:500 (14-9965, eBioscience); anti-Sox2, 1:200 (sc-17320, Santa Cruz); anti-S100β, 1:200 (S2657-0.2ML, Sigma-Aldrich); anti-BrdU, 1:500 (ab6326, Abcam); anti-cleaved caspase3, 1:500 (9664S, Cell Signaling Technology); and anti-GFAP, 1:1000 (ab4674, Abcam). Images were acquired with a laser‐scanning confocal microscope (TSC‐SP5, Leica or LSM 880, Zeiss) and processed with ImageJ software (NIH).

### Immunohistofluorescence analysis

Immunohistofluorescence analysis was performed as described previously^69^. Adult (P56–P63) B6J mice were subjected to perfusion fixation with 4% paraformaldehyde in PBS, and the brain was dissected and fixed for 24 h with the same solution. E14.5 or E16.5 B6J embryos were fixed for 2 h with the same fixative. The isolated brain subjected to cryoprotection with sucrose and embedded and frozen in OCT compound (Tissue TEK). Sections prepared at a thickness of 10–12 μm were exposed to 0.1% Triton X‐100 and 3% bovine serum albumin in Tris‐buffered saline (blocking solution) for 1 h at room temperature before incubation first overnight at 4 °C with primary antibodies diluted in blocking solution and then for 1 h at room temperature with Alexa Fluor–labeled secondary antibodies (1:1000 dilution) (A11055, A11039, A31572, A31570, A31571, A21447, Molecular Probes) and Hoechst 33342 (1:1000 dilution) (Molecular Probes) also diluted in blocking solution. They were finally mounted in Mowiol (Calbiochem) for imaging with a laser‐scanning confocal microscope (LSM 880, Zeiss), and images were processed with ImageJ software (NIH). Primary antibodies and their dilutions were as follows: anti-Id1, 1:500 (BCH-1/37-2, Biocheck); anti-Id4, 1:500 (BCH-9/82-12, Biocheck); anti-S100, 1:200 (ab4066 4C4.9, Abcam); anti-GFAP, 1:1000 (ab4674, Abcam); anti-Sox2, 1:200 (sc-17320, Santa Cruz); and anti-Ascl1, 1:200 (556604, BD Pharmingen).

### Immunoblot analysis

Immunoblot analysis was performed as described previously^70^. Brain tissue or cultured cells were lysed with a solution containing 50 mM Tris–HCl (pH 7.5), 150 mM NaCl, 1% NP-40, 1 mM EDTA, 0.1% sodium deoxycholate, 0.1% SDS, 1 mM dithiothreitol, and aprotinin (1 mg/ml). Phosphatase inhibitor (PHOSS-RO, Sigma-Aldrich) was added to the solution according to the manufacture’s instruction. The protein concentration of the lysates was determined with a Pierce BCA Protein Assay Kit (Thermo Fisher Scientific). Protein samples (~ 10 μg) were fractionated by SDS–polyacrylamide gel electrophoresis on a 8–16% gel (Biorad) and the separated proteins were transferred to a polyvinylidene difluoride membrane (Millipore), which was then exposed for 1 h at room temperature to 2% dried nonfat milk before incubation overnight at 4 °C with antibodies to phospho-Smad1/5/8 (D5B10, Cell Signaling Technology, 1:500), to Id1 (BCH-1/37-2, Biocheck, 1:500), to Id4 (BCH-9/82-12, Biocheck, 1:500), to Foxj1 (HPA005714, Sigma-Aldrich, 1:500) or to GAPDH (MAB374, Sigma-Aldrich, 1:2000). The membrane was then incubated for 1 h at room temperature with horseradish peroxidase–conjugated secondary antibodies (GE Healthcare), after which immune complexes were detected with a chemiluminescence reagent [100 mM Tris–HCl (pH 8.5), 1.25 mM luminol, 0.2 mM coumaric acid, 0.009% H_2_O_2_ or Super Signal West Atto Ultimate Sensitivity Substrate (Thermo Fisher Scientific)]. The images were acquired by Fusion instrument (M&S Instrument Inc.) and quantified using Evolution Capt software (M&S Instrument Inc).

### Statistical analysis

Quantitative data are presented as means ± SEM and were compared either among multiple groups with one-way analysis of variance followed by Tukey’s post hoc test or between two groups with the two-tailed Student’s paired or unpaired *t* test. A *p* value of < 0.05 was considered statistically significant. The number of animals in each experiment is stated in the respective figure legends.

## Supplementary information

Supplementary Figures.
