# Supplementary material for: BMP signaling suppresses Gemc1 expression and ependymal differentiation of mouse telencephalic progenitors
Source: Sci Rep. 2021 Jan 12;11:613. doi: 10.1038/s41598-020-79610-6 (PMC7804439; doi:10.1038/s41598-020-79610-6)
Supplement: Supplementary file 1 — Supplementary Figures. [file 41598_2020_79610_MOESM1_ESM.docx]

**Supplementary Information**

**BMP signaling suppresses *Gemc1* expression and ependymal differentiation of mouse telencephalic progenitors**

Hanae Omiya^1^, Shima Yamaguchi^1^, Tomoyuki Watanabe^1^, Takaaki Kuniya^1^,

Yujin Harada^1^, Daichi Kawaguchi^1^*, Yukiko Gotoh^1,2^*

^1^Graduate School of Pharmaceutical Sciences, The University of Tokyo, Tokyo 113-0033, Japan

^2^International Research Center for Neurointelligence (WPI-IRCN), The University of Tokyo, Tokyo 113-0033, Japan

*Correspondence: Daichi Kawaguchi, Ph.D., Graduate School of Pharmaceutical Sciences, The University of Tokyo, 7-3-1 Hongo, Bunkyo-ku, Tokyo 113-0033, Japan. Tel.: +81-3-5841-4870. Email: [dkawaguchi@mol.f.u-tokyo.ac.jp](mailto:dkawaguchi@mol.f.u-tokyo.ac.jp); Yukiko Gotoh, Ph.D., Graduate School of Pharmaceutical Sciences, IRCN, The University of Tokyo, 7-3-1 Hongo, Bunkyo-ku, Tokyo 113-0033, Japan. Tel.: +81-3-5841-4870. Email: ygotoh@mol.f.u-tokyo.ac.jp


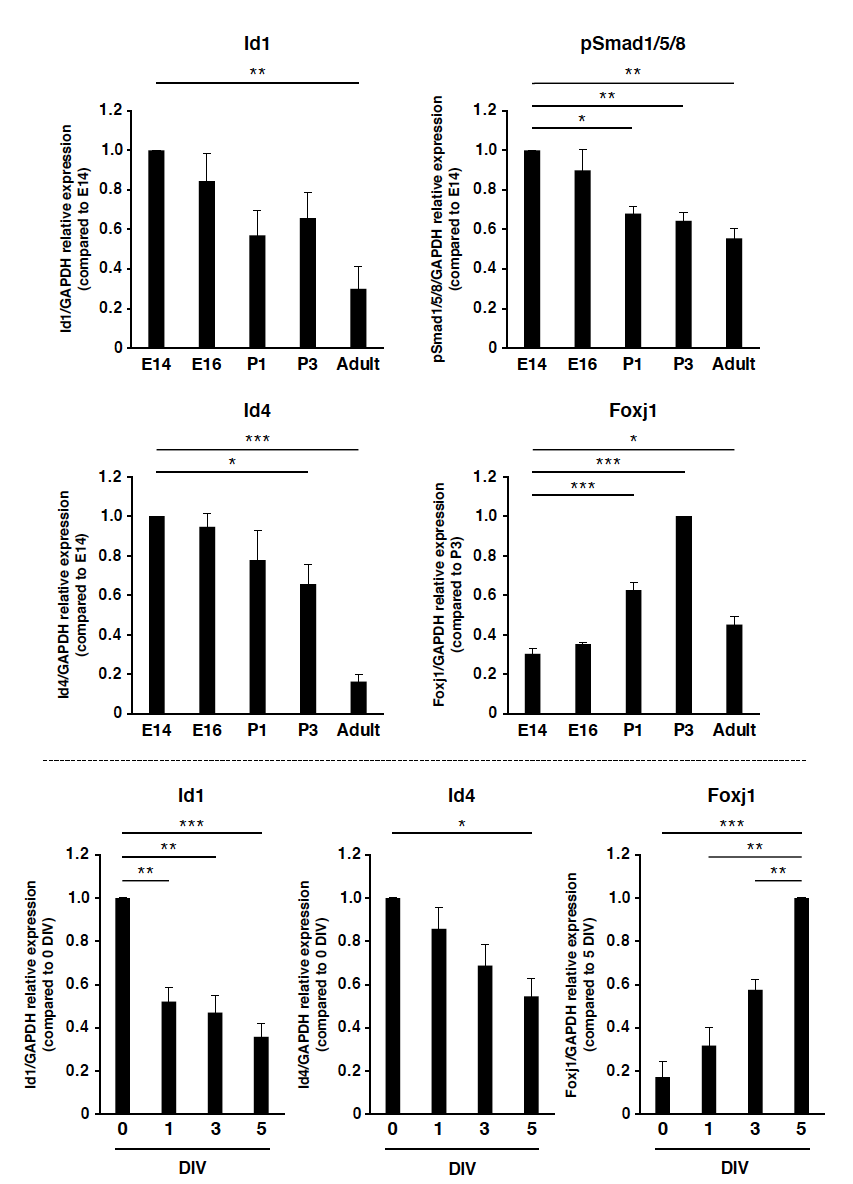


**Supplementary Figure 1.** Quantification of the corresponding bands in the immunoblot analyses shown in Figure 1c (upper panels) and 1e (lower panels). Data are means ± SEM (n=3 independent experiments). *p < 0.05, **p < 0.01, ***p < 0.001 (Tukey’s test).


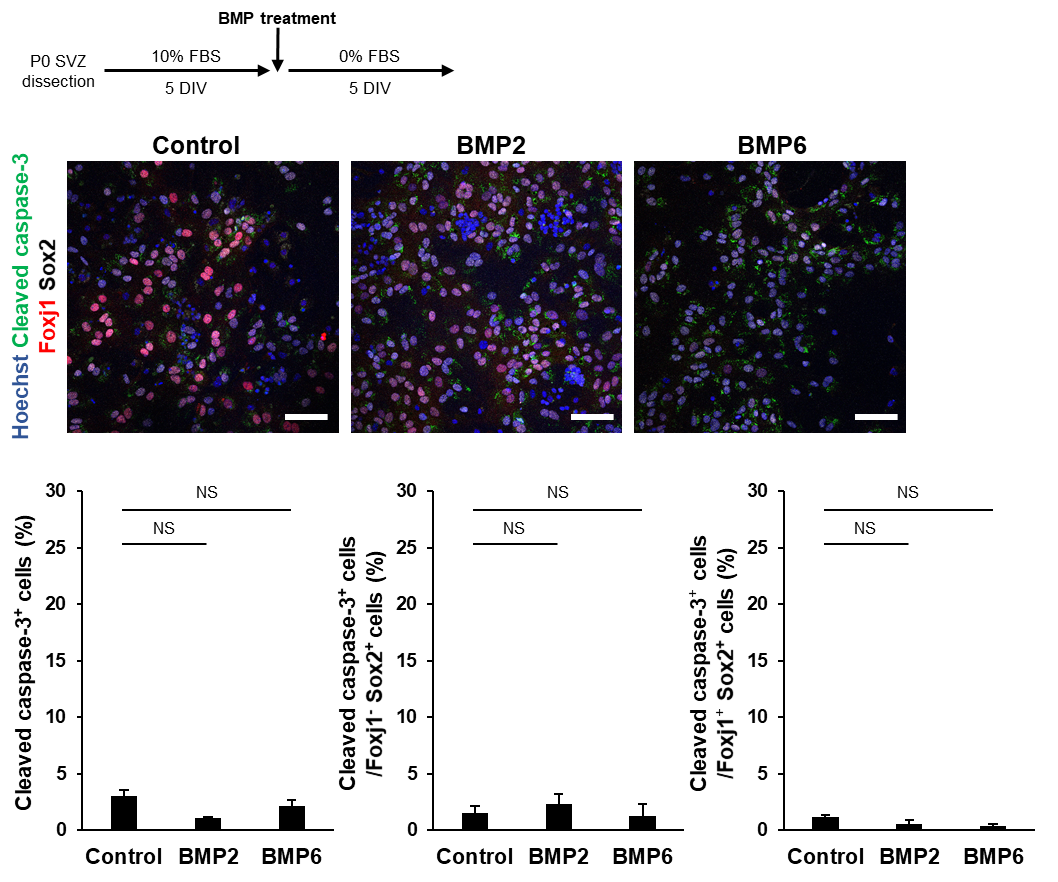


**Supplementary Figure 2.** Evaluation of activated caspase-3 in the BMP treated postnatal NPC culture. NPC cultures prepared at P0 and maintained for 5 days in the presence of 10% FBS and then for 5 days with 0% FBS in the absence or presence of BMP2 (20 ng/ml) or BMP6 (20 ng/ml) were subjected to immunocytofluorescence staining with antibodies to Foxj1, Sox2 and to cleaved caspase-3. Nuclei were counterstained with Hoechst 33342. Scale bars, 50 μm. The percentage of cells with nuclear signals of cleaved caspase-3 among Foxj1^-^Sox2^+^ NPCs or among Foxj1^+^Sox2^+^ ependymal cells was determined. Three microscope fields were counted per sample. Total of 300~500 cells were counted per sample. Data are means ± SEM (n=3 independent experiments). NS = not significant (Tukey’s test).

**Supplementary Figure 3.** Evaluation of BrdU incorporation in the BMP treated postnatal NPC culture. NPC cultures prepared at P0 were maintained for 5 days in the presence of 10% FBS and for 1 day with 0% FBS in the absence or presence of BMP2 (20 ng/ml) or BMP6 (20 ng/ml) before treatment with BrdU (10 μM) for 2 h. These cells were subjected to immunocytofluorescence staining with antibodies to Foxj1, Sox2 and to BrdU. Nuclei were counterstained with Hoechst 33342. Scale bars, 50 μm. The percentage of BrdU^+^ cells among Foxj1^-^Sox2^+^ NPCs or among Foxj1^+^Sox2^+^ ependymal cells were determined. Three microscope fields were counted per sample. Total of 300~500 cells were counted per sample. Data are means ± SEM (n=3 independent experiments, Tukey’s test).


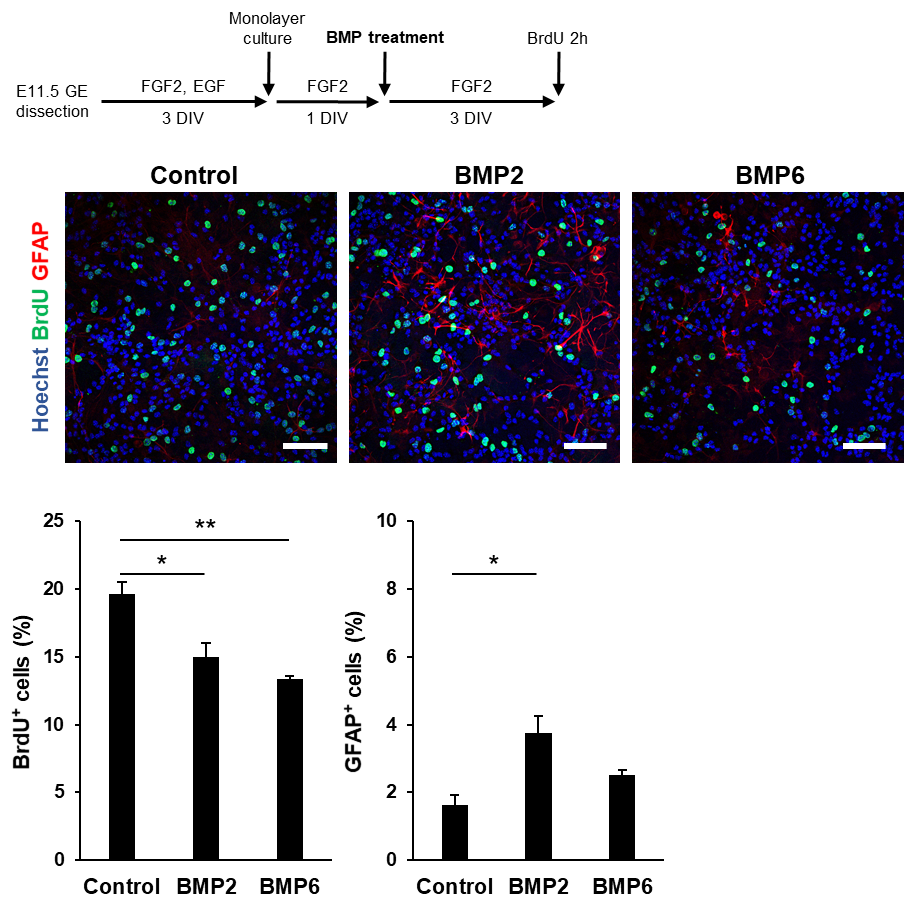


**Supplementary Figure 4.** BMP treatment increased GFAP^+^ cells and reduced BrdU^+^ cells in the embryonic NPC culture. Cells prepared from the GE of E11.5 embryos were cultured for 3 days in suspension, and the resulting neurospheres were dissociated and cultured as monolayers for 1 day before exposure to BMP2 (50 ng/ml) or BMP6 (50 ng/ml) for 3 days before treatment with BrdU (10 μM) for 2 h. Cultures were subjected to immunocytofluorescence staining with antibodies to BrdU, Sox2 and to GFAP. The percentages of BrdU^+^ cells or GFAP^+^ cells were determined. Nuclei were counterstained with Hoechst 33342. Scale bars, 50 μm. Three microscope fields were counted per sample. Total of 200~300 cells were counted per sample. Data are means ± SEM (n=3 independent experiments). *p < 0.05, **p < 0.01 (Tukey’s test).

**Supplementary Figure 5.** Full length original scanned images of the immunoblot analysis in Figure 1c and e.
